# Silica Aerogel-Polycaprolactone Scaffolds for Bone Tissue Engineering

**DOI:** 10.3390/ijms241210128

**Published:** 2023-06-14

**Authors:** Ana Dora Rodrigues Pontinha, Beatriz Barbosa Moreira, Bruna Lopes Melo, Duarte de Melo-Diogo, Ilídio Joaquim Correia, Patrícia Alves

**Affiliations:** 1 University of Coimbra, CIEPQPF, Department of Chemical Engineering, Faculty of Sciences and Technology, Rua Sílvio Lima, 3030-790 Coimbra, Portugal; 2 University of Coimbra, ISISE, Department of Civil Engineering, Rua Luís Reis Santos, 3030-788 Coimbra, Portugal; 3 CICS-UBI, Centro de Investigação em Ciências da Saúde, Universidade da Beira Interior, 6200-506 Covilhã, Portugal

**Keywords:** hybrid composites, poly-ε-caprolactone (PCL), silica aerogel, tissue engineering

## Abstract

Silica aerogel is a material composed of SiO_2_ that has exceptional physical properties when utilized for tissue engineering applications. Poly-ε-caprolactone (PCL) is a biodegradable polyester that has been widely used for biomedical applications, namely as sutures, drug carriers, and implantable scaffolds. Herein, a hybrid composite of silica aerogel, prepared with two different silica precursors, tetraethoxysilane (TEOS) or methyltrimethoxysilane (MTMS), and PCL was synthesized to fulfil bone regeneration requirements. The developed porous hybrid biocomposite scaffolds were extensively characterized, regarding their physical, morphological, and mechanical features. The results showed that their properties were relevant, leading to composites with different properties. The water absorption capacity and mass loss were evaluated as well as the influence of the different hybrid scaffolds on osteoblasts’ viability and morphology. Both hybrid scaffolds showed a hydrophobic character (with water contact angles higher than 90°), low swelling (maximum of 14%), and low mass loss (1–7%). hOB cells exposed to the different silica aerogel-PCL scaffolds remained highly viable, even for long periods of incubation (7 days). Considering the obtained results, the produced hybrid scaffolds may be good candidates for future application in bone tissue engineering.

## 1. Introduction

Aerogels are advanced nanostructured materials discovered in the 1930s [1] and well-known by their three-dimensional nanoporous structure [2,3,4,5]. Due to their characteristic high specific surface area with low density, aerogels are attractive to a variety of fields, including the biomedical and pharmaceutical ones [6,7,8]. These materials are usually prepared by sol–gel processes [3,9,10], which promote, at low temperatures, the synthesis of their network by chemical reactions in solution [4,11]. The resulting solid material is ultralightweight, porous and, in some cases, transparent, comprising 99.2% of empty space [3].

Aerogels are classified into organic, inorganic, and hybrid aerogels according to the type of precursor used during their synthesis [3,12]. However, according to their surface chemical properties, they are categorized into hydrophobic or hydrophilic aerogels [13].

Recently, aerogel materials have attracted a great attention for biomedical applications. In this regard, aerogels have been explored in different applications, such as tissue engineering, drug/protein delivery, bone grafting, biosensing, and blood sorption [8,14,15].

Due to their high water uptake, interconnected porous structure, and excellent permeability, aerogels are promising candidates to be applied for scaffolds production [16,17,18].

In addition, aerogels have also been combined with different polymers for the fabrication of porous 3D scaffolds [19] aimed for tissue engineering applications [20,21,22,23]. Current research on biopolymer-based aerogels is motivated by the exploration of safer, less toxic, and more sustainable precursors with better properties for the preparation of delivery systems [15]. The hybrid polymeric composites have been widely explored due to their ability to combine the advantages of a polymeric matrix with those of inorganic components and exhibit better properties than the pure counterparts [16]. Other works have been performed with the intent to achieve improvements in supporting cell migration and tissue regeneration, modifying the pore size and radius of curvature of the tissue engineering scaffold [24]. Therefore, aerogel-polymer hybrid scaffolds combine the advantages of both technologies, outperforming their pure counterparts.

Poly-ε-caprolactone (PCL) is a biodegradable polymer and member of the aliphatic polyester family [25]. This polymer possesses several important features such as benign biocompatibility, low cost, biodegradability, and easy fabrication [26,27]. The combination of PCL with other biopolymers enhances the properties of the resultant scaffolds [28,29,30,31]. PCL is a good candidate for tissue engineering in terms of cells’ attachment, matrix production, and proliferation [32,33,34]. Previous studies have demonstrated positive effects of PCL composites on osteoblasts when used as a bone graft substitute [34,35,36]. PCL has also been studied for reconstruction of many other tissues, including nerves and cartilage [37].

Silica aerogel has already been combined with PCL as tissue engineering scaffold, however few reports are available [17,38]. For instance, Ge et al. [38] prepared silica aerogel/PCL membranes using silica aerogel powders and PCL. Their results demonstrated that the basic silica aerogel neutralizes the scaffolds and promotes cell survival and growth. Goimil et al. [17] reported the effects of the incorporation of aerogels, using supercritical technologies (a green but expensive process), in the properties of synthetic PCL-based scaffolds. Moreover, the characterization of these silica aerogel/PCL properties in animal models for bone and cartilage tissue repair/engineering, and the cellular and molecular mechanisms involved in the regulation of cell survival and proliferation under the presence of the biomaterial components, are limits of these works.

In this research work, hybrid scaffolds were produced, and their properties evaluated. The goal was to develop a biocompatible scaffold with suitable mechanical properties, without the need of the addition of phosphates or other inorganic compounds. These hybrid scaffolds were obtained from silica-aerogel and PCL which could be applied in tissue engineering, particularly as bone grafting materials. To accomplish this, PCL was hybridized with two different silica precursors, one hydrophilic and another hydrophobic, tetraethoxysilane (TEOS) and methyltrimethoxysilane (MTMS), respectively. These porous hybrid scaffolds were fabricated by adding aerogel, obtained by the sol–gel process, to PCL. The hybrid scaffolds were then extensively studied in terms of thermal and physico-chemical properties and water absorption capacity. The influence of the different hybrid scaffolds on osteoblasts’ viability and morphology was also assessed.

## 2. Results and Discussion

The main goal of this work was to produce a biomaterial composed of PCL and aerogel suitable for tissue engineering applications. To check the suitability of the produced biomaterials for the aimed biomedical application, different characterization techniques were used.

### 2.1. Chemical Characterization

**Figure 1:**
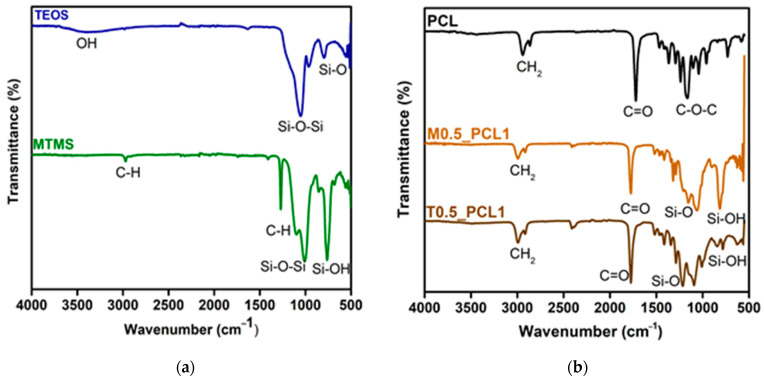
FTIR/ATR spectra of (**a**) TEOS and MTMS aerogel, and (**b**) PCL and M0.5_PCL1 and T0.5_PCL1 samples.

The ATR-FTIR spectra of the different components of the TEOS-based aerogel, MTMS-based aerogel, PCL, and composites M0.5_PCL1 and T0.5_PCL1 are presented in Figure 1.

In the spectrum corresponding to TEOS-based aerogel (Figure 1a), the bands that demonstrate the formation of the silica structure are the Si–O symmetrical elongation vibration observed at 800 cm^−1^ and the Si–O–Si asymmetric elongation vibration (≈1080–1200 cm^−1^ range). This last Si–O–Si bond in the silica aerogel was observed at 1070 cm^−1^, which confirmed the formation of TEOS-based aerogels [39].

The formation of MTMS-based aerogels is validated by the presence of bands at 1030–1110 cm^−1^ which are attributed to the Si–O–Si asymmetrical stretch vibration, the bands at 1272 cm^−1^ and 1409 cm^−1^ which are attributed to stretch vibration, and the C–H absorption peak, which is consistent with previous research results [40]. The Si–OH stretch vibration band at 919 cm^−1^ indicates the presence of more residual silanol groups. Vibrations at the 1350–1500 cm^−1^ and 2800–3000 cm^−1^ range correspond to C–H bonds.

By analyzing the PCL spectrum (Figure 1b), bands at 2943 and 2865 cm^−1^ correspond to the CH_2_ asymmetric stretching and CH_2_ symmetric stretching, respectively. The carbonyl stretching (C=O) appears at 1727 cm^−1^ and the stretching C–O and C–C in the crystalline phase at 1292 cm^−1^. The bands at 1237 and 1167 cm^−1^ are in line with the asymmetric C–O–C stretching and symmetric C–O–C stretching, respectively [41].

The FTIR spectra of composites M0.5_PCL1 and T0.5_PCL1 showed bands similar to the ones presented in the pure PCL spectrum. The main differences are observed in the peaks at wavelengths of 1080, 1200, and 800 cm^−1^ corresponding to the asymmetric and symmetric Si–O stretching vibrations of the aerogels. Si–O–Si bond bending vibrations and Si–OH bond vibrations were attributed to peaks at 1090 cm^−1^ and 560 cm^−1^, respectively [42]. The presence of both PCL and silica peaks suggests that the composites present both materials in their structure (further analyzed in the next sub-section).

### 2.2. Surface Morphology

The determination of the specific surface areas of the composites was based on the nitrogen adsorption isotherms and the obtained values are shown in Table 1. The samples show lower specific surface area values than those for other aerogels composites [43]. The lower specific surface area can be related to the presence of non-porous PCL, creating large interstices in the material.

TEOS-based aerogel composites showed a lower surface area, which implies a more compact composite. The MTMS-based aerogel composite presents the higher specific surface area explained by the presence of CH_3_ groups in the structure.

**Figure 2:**
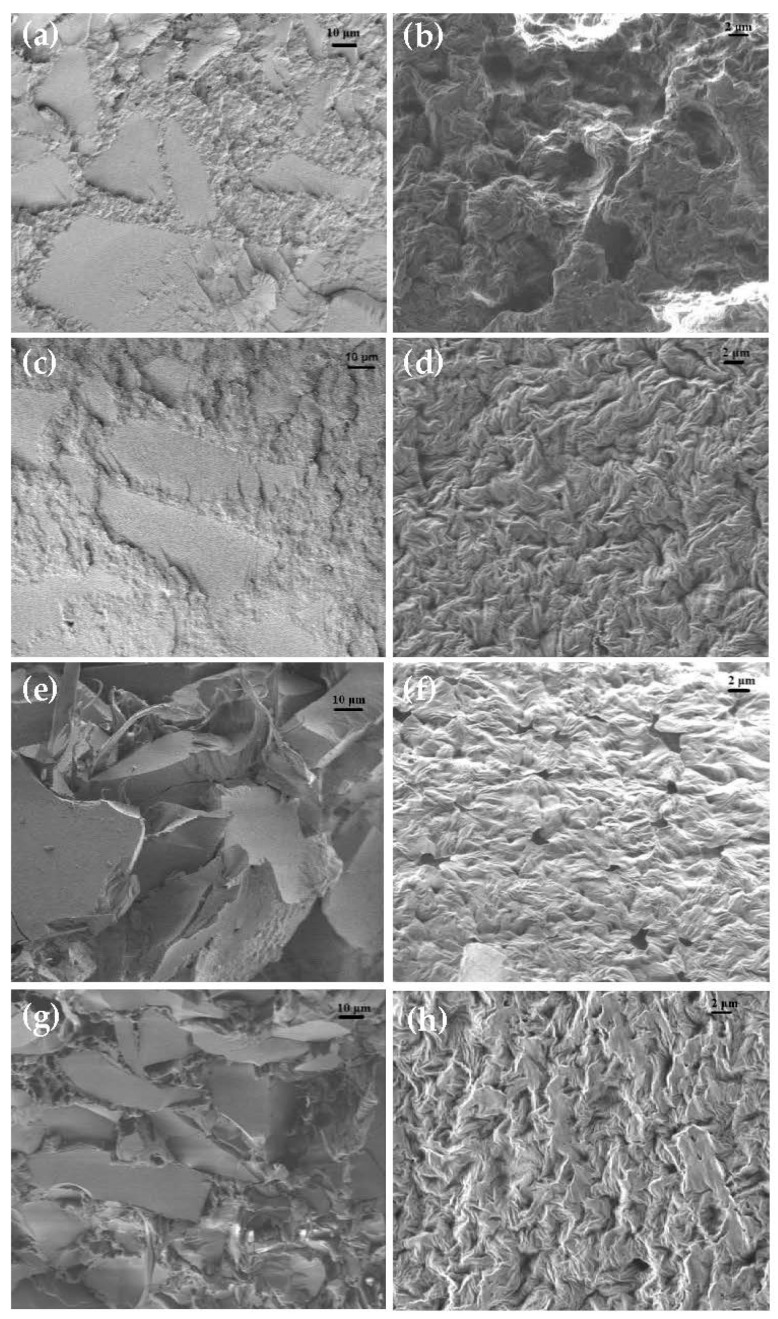
Cross-section (left) and surface (right) and scanning electron microscopy (SEM) images of the following composites: (**a**,**b**) M0.5_PCL1; (**c**,**d**) M0.25_PCL1; (**e**,**f**) T0.5_PCL1; and (**g**,**h**) T0.25_PCL1.

To better understand the morphology of the composites, surface and cross-section scanning electron microscopy (SEM) analysis were performed and the obtained images are displayed in Figure 2.

Through the analysis of the SEM surface images, it is possible to observe that the MTMS composites (Figure 2b,d) present a rough and irregular surface with some shapes that might induce a porous morphology in the entire surface, which corroborates the higher porosity obtained by BET analysis, along with smoother zones that are related to the silica-aerogel particles (shown in EDS results). In turn, TEOS composites (Figure 2f,h) show an evident irregular, less porous surface. T0.25_PCL composites (Figure 2h) present a rougher surface when compared to T0.5_PCL1 composite surfaces and a less evident porosity.

Analyzing the cross-sections, it is possible to observe that M0.5_PCL1 composite (Figure 2a) suffered a brittle fracture, due to the immersion in liquid nitrogen employed to break the samples. This is similar to the M0.25_PCL1 composite (Figure 2c), where the smooth and square surface structures correspond to silica along with the presence of some porosity. T0.5_PCL1 composite (Figure 2f), on the other hand, suffered a ductile fracture (irregular appearance; it suffered plastic deformation before the fracture). Here, it is easier to distinguish the silica from the polymer and that the surfaces angular shapes are silica. T0.25_PCL1 composite (Figure 2g) shows a less ductile fracture than the previous sample. For all samples, it is possible to observe that there is some porosity, that the silica is well incorporated with the polymer, and that it is homogeneously distributed. 

**Figure 3:**
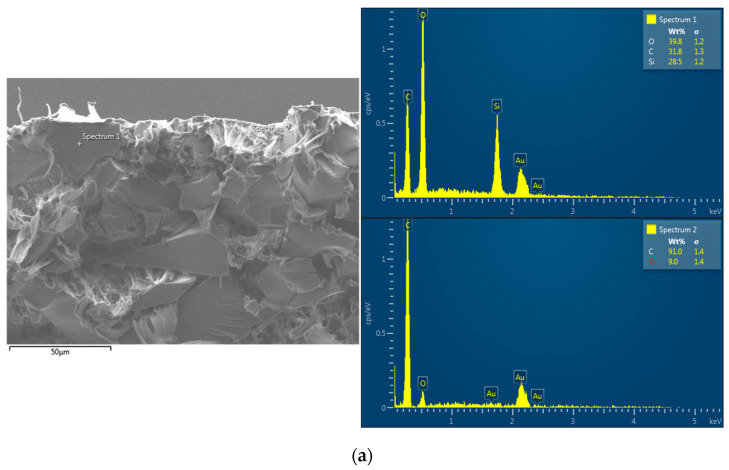

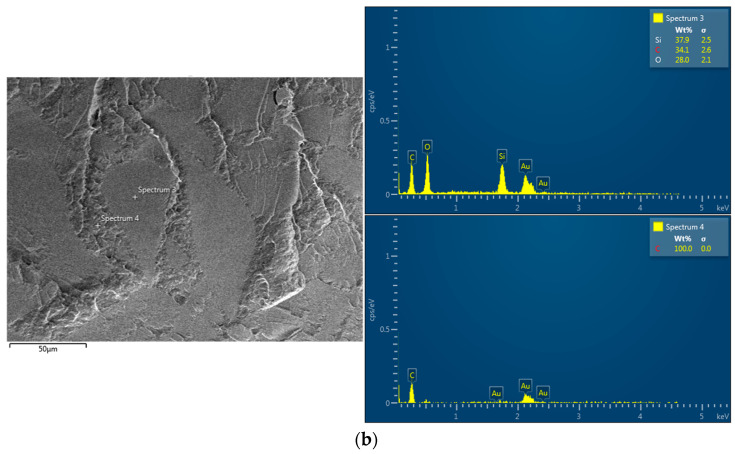
EDS analysis of (**a**) T0.25_PCL1 and (**b**) M0.5_PCL1.

EDS analysis was carried out to confirm if the smooth, irregular, and square surfaces observed in the cross-sectioned samples were the silica particles mixed with PCL. Figure 3 shows that the smooth sections were indeed from the aerogel and that the samples with a ratio of 0.5:1 (silica aerogel: PCL) actually have a higher silica content (37.9%) than the samples with 0.25:1 (silica aerogel: PCL, 28.5%). In general, it can be said that the silica-aerogel particles are well-distributed throughout the bulk of the scaffold, with polymeric transition zones whose composition seems to be only polymeric.

### 2.3. Surface Hydrophobicity

Surface hydrophilicity/hydrophobicity was evaluated by water contact angle determination on the surface of the different obtained composites. This is an important parameter since it gives information regarding the interaction of the composites with the surrounding environment, such as cells, other materials, or moisture. Table 2 shows the values obtained for the static water contact angle, which varied between 90° and 100°.

The results obtained confirmed the hydrophobic character of the composites, presenting a contact angle value higher than 90° for all samples. This is due to the composition of samples: PCL is a hydrophobic polymer, with a water contact angle around 140° [44], and aerogels have in their composition the presence of non-polar methyl groups (Si–CH_3_) [45]. However, since TEOS aerogels are known to be less hydrophobic when compared to MTMS-based aerogel due to the presence of some hydroxyl groups in their structure (as shown by the ATR-FTIR analysis), the composites with TEOS aerogels show slightly lower water contact angle. 

### 2.4. Bulk Density 

The bulk density of the prepared composite samples was determined and is presented in Table 3. As reported in the literature, silica aerogels are known for their very low density (0.003–0.15 kg/m^3^) [3]. On the other hand, PCL presents itself with a high density (~1145 kg/m^3^). Moreover, according to the literature, the natural bone density is 1700–20,000 kg/m^3^ [46,47,48]. According to the results presented in Table 2, composites with a silica/PCL ratio of 0.5:1 showed lower density values than those of 0.25:1; this behavior is expected due to an increase in silica when compared with the former composite. Moreover, it is possible to observe that the composites with MTMS and PCL have higher density than those of TEOS with PCL, probably due to the presence of some hydroxyl groups, which improve wettability (already shown by water contact angles measurements) and by the more porous surface shown in SEM images.

### 2.5. Thermal Properties

Thermogravimetry analysis (TGA) was performed to evaluate the thermal stability of the materials used. Regarding the thermal stability of these materials—aerogel, PCL, and aerogel-PCL composites—they remain stable up to approximately 350 °C. 

**Figure 4:**
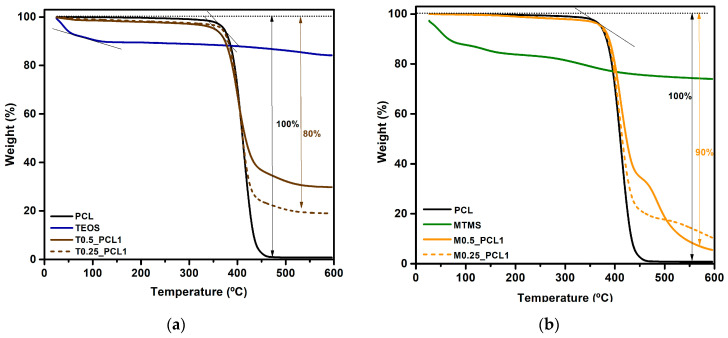
TGA traces of (**a**) PCL, TEOS-based aerogel, T0.5_PCL1 and T0.25_PCL1 samples; (**b**) PCL, MTMS-based aerogel, M0.5_PCL1 and M0.25_PCL1 samples.

PCL TGA shown in Figure 4 displays a slight mass loss around 100 °C, resulting from the loss of residual water present in the sample. PCL showed a good thermal stability, starting to degrade only at 360 °C, and presented only one step of degradation. As already reported [49], PCL decomposes in nitrogen atmosphere at approximately 350 °C to 5-hexenioc acid and carbon dioxide, and at temperatures > 430 °C, ε-caprolactone is evolved.

TEOS-aerogel based TGA, Figure 4a, shows a weight loss near 60 °C. This is the result of the evaporation of entrapped H_2_O and alcoholic groups from less hydrophobic silica aerogels as a result of the condensation reactions of Si–OH and Si(OC_2_H_5_) groups [43]. 

MTMS-based aerogel TGA, Figure 4b, indicates that the process of weight change could be initially caused by the loss of ethanol solvent and other volatile organic materials. The sharp mass decline observed at 375–550 °C was caused by the release of methyl (-CH_3_) groups from the MTMS aerogel structure because the oxidation reaction formed CO_2_ and the oxidation of Si-C group became Si-O, which further formed a siloxane group [50].

The different composites, T0.5_PCL1, T0.25_PCL1, M0.5_PCL1, and M0.25_PCL1, had their behavior influenced by both PCL and aerogel (TEOS or MTMS). The TGA curves show mass losses ranging from 70% to 90% for these composites.

### 2.6. Mechanical Properties

The main purpose of this composite material is to be used as a scaffold in bone regeneration. Polymeric scaffolds have significant advantages over metals and ceramics in terms of biodegradability. However, the compressive strength of the implant is usually lower than the native bone tissue. The mechanical properties of cortical and cancellous bone are difficult to measure and tend to vary depending on bone orientation. The elastic modulus of cortical bone is approximately in the range of 3–30 GPa [51,52], and the elastic modulus of cancellous bone is estimated to be 0.02–6 GPa [53,54].

**Figure 5:**
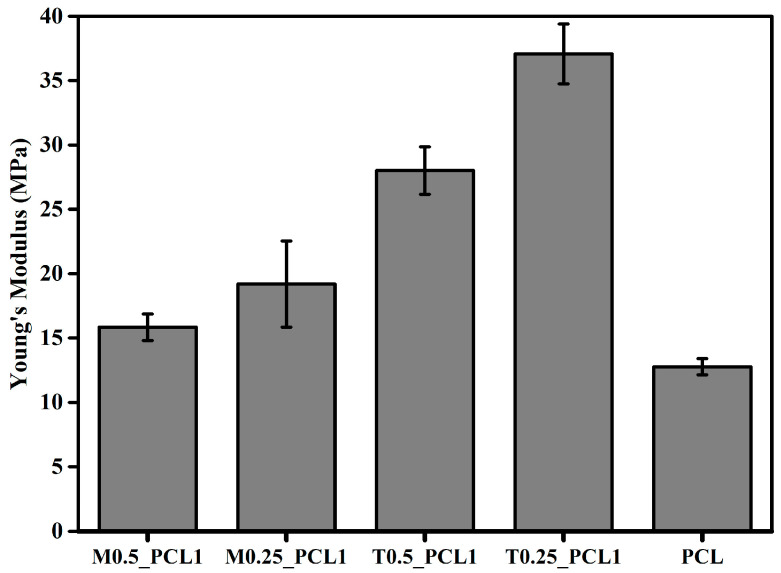
Young’s modulus obtained in tensile mechanical tests, for the different composites (*n* = 4): M0.5_PCL1, M0.25_PCL1, T0.5_PCL1, T0.25_PCL1, and PCL, using a cell with a maximum load of 3 kN.

The mechanical behavior of the different samples was obtained by tensile tests under the same conditions, using a 30 kN cell, and the obtained results are shown in Figure 5. The effect of the amounts of silica aerogel on the mechanical properties of PCL was investigated.

Figure 5 shows that PCL is the less rigid material, showing the lowest Young’s modulus (*Y*_M_ = 12.77 ± 0.63 MPa) given its elastomeric behavior. The addition of the silica aerogel leads to an increase in Young’s modulus, because of the material’s higher stiffness. The addition of silica is important to introduce pore structures in the composite. These porous structures are required in scaffolds for a good vascularization and bone regeneration subsequent to implantation [55].

PCL is a hemihedral crystal, with low Young’s modulus due to the fact that its macromolecular chains has about 45% crystallinity [56]. The introduction of silica aerogel leads to an increase in the Young’s modulus, increasing the stiffness of the composite. The C-C and C-O bonds present in the structure of PCL are less rigid than Si-O-Si siloxane bonds. 

Young’s modulus values obtained are lower than those mention previously for cortical bone (3–30 GPa [51,52]) but are within the range of the values for cancellous bone 0.02–6 GPa [53,54]. It is also important to mention that Young’s modulus increases when switching from MTMS-based aerogel to TEOS-based aerogel, as shown in Table 4. The CH_3_ bonds present in the MTMS aerogel led to a lower Young’s modulus due to the flexibility of this group [43] and also the free volume effects. This agrees with the results in Table 1, where MTMS-based aerogel composites have higher specific surface area, and therefore are less rigid than TEOS-based aerogel composites. Regarding the maximum stress, the presence of TEOS-based aerogel causes an increase relativity to the PCL samples. This increase could be related to the more facility alignment of the PCL chains and the resulting bigger intermolecular interactions between the PCL chains that occur during regeneration and water evaporation.

### 2.7. Swelling and Degradation

**Figure 6:**
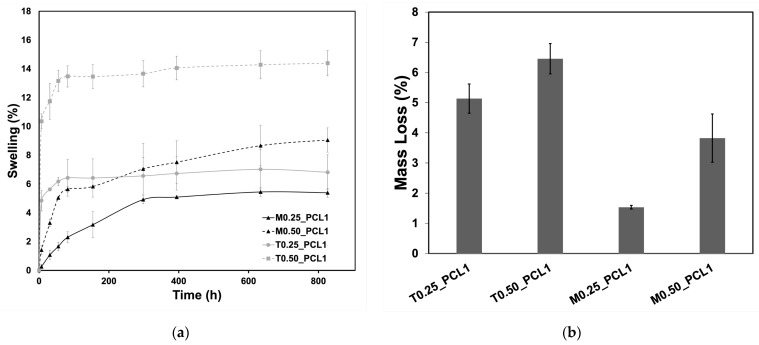
Swelling profile (**a**) and mass loss (**b**) of the hybrid scaffolds (*n* = 4).

The swelling capacity and the average total mass loss of each tested scaffold are represented in Figure 6a,b, respectively. Generally, the hybrid scaffolds showed low swelling (maximum of 14%) and low mass loss; this behavior is expected considering their hydrophobic character and the low degradability of both aerogel and PCL components. Despite the scaffolds’ porosity, shown by SEM, their low affinity to water limits its intake and therefore the swelling, as well as the degradation of the samples. Since TEOS-based aerogel scaffolds are slightly less hydrophobic than MTMS-based aerogel scaffolds, the latter showed lower degradation and swelling. Moreover, samples presented a similar swelling profile, characterized by an increased water uptake in the first hours leading to a plateau. In this regard, the T0.50_PCL1 scaffold presented the highest swelling, which may be correlated with its low hydrophobicity and density, leading to a higher mass loss, while a lower proportion of silica, namely T0.25_PCL1 and M0.25_PCL1, led to lower swelling capacity and lower mass loss.

### 2.8. Cytocompatibility

**Figure 7:**
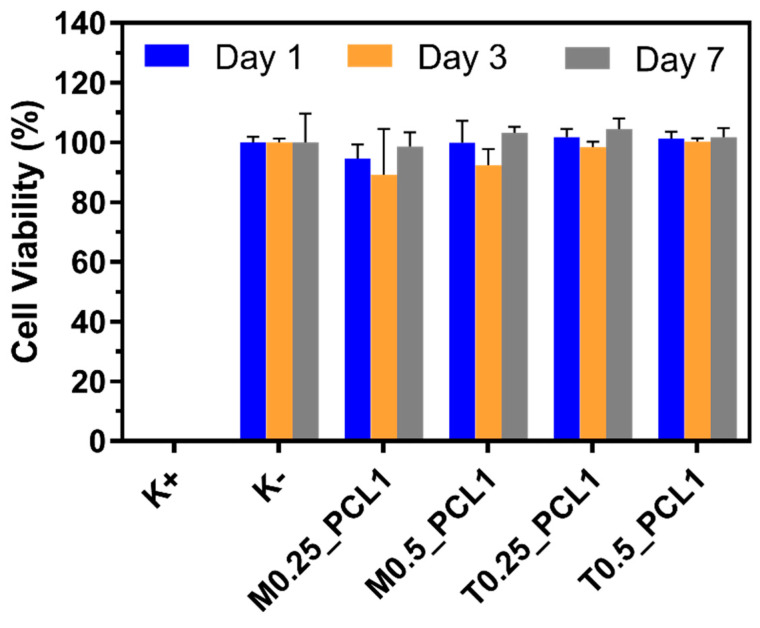
Viability of hOB cells exposed to M0.25_PCL1, M0.5_PCL1, T0.25_PCL1, and T0.5_PCL1 for 1, 3, and 7 days. Data represent mean ± standard deviation, *n* = 5. K− and K+ denote the negative and positive controls, respectively.

The cytocompatibility of the produced silica aerogel-PCL scaffolds towards hOB cells was assessed through the resazurin assay (Figure 7). The obtained results suggest that the hOB cells exposed to the different silica aerogel-PCL scaffolds remained highly viable, even for long incubation periods of 7 days (Figure 7).

**Figure 8:**
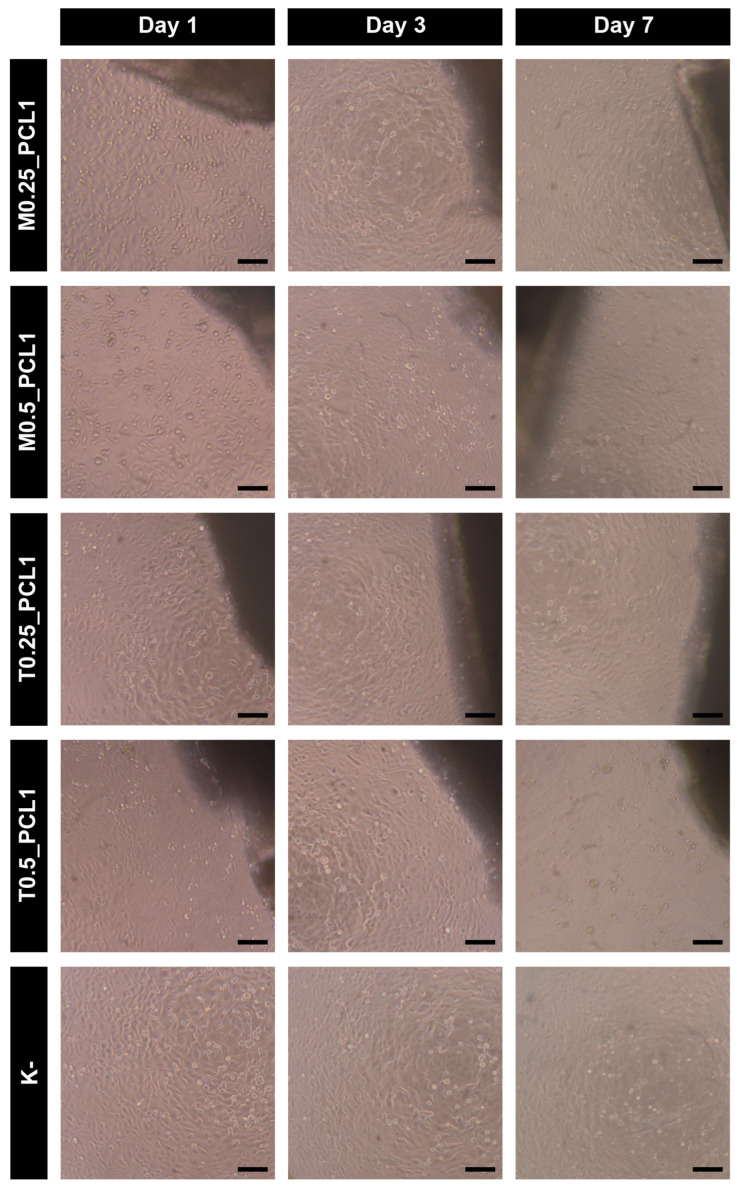
Optical microscopy images of hOB cells incubated with M0.25_PCL1, M0.5_PCL1, T0.25_PCL1, and T0.5_PCL1 for 1, 3, and 7 days. K− denotes the negative control. The scale bar represents 100 μm.

Nevertheless, the optical microscopy images highlighted a differential behavior induced by the tested materials. In agreement with the cell viability data, the hOB cells in close proximity with the M0.25_PCL1 and T0.25_PCL1 displayed a similar density and morphology to the control along the 7 days of incubation (Figure 8). However, for the M0.5_PCL1 and T0.5_PCL1, this similarity to the control was only observed for the incubation periods of 1 and 3 days (Figure 8). In fact, hOB cells in close proximity with M0.5_PCL1 appeared to present a slightly lower density after 7 days of incubation, while cells exposed to T0.5_PCL1 for this same period displayed changes on both density and morphology (Figure 8). Altogether, these results highlight that M0.25_PCL1 and T0.25_PCL1 present enhanced biological properties when compared to the other two formulations (M0.5_PCL1 and T0.5_PCL1).

Ge et al. [37] also produced hybrid scaffolds of silica aerogels and PCL that appeared to be cytocompatible towards 3T3 cells during 4 days of incubation. However, extending the incubation time for 7 days revealed a cytotoxic behavior. Herein, the produced M0.25_PCL1 and T0.25_PCL1 scaffolds revealed a good cytocompatiblity at all the tested time points (1, 3, and 7 days of incubation), suggesting an enhanced biological performance and that the obtained hybrid scaffolds may be good candidates for bone tissue engineering applications.

## 3. Materials and Methods

### 3.1. Materials

For composites production, tetraethoxysilane (TEOS, purity ≥ 98%, Acros organics, (Porto Salvo, Portugal), Si(OC_2_H_5_)_4_); methyltrimethoxysilane (MTMS, purity ≥ 98%, Sigma-Aldrich (Sintra, Portugal), Si(OCH_3_)_3_CH_3_); peracetic acid (38–40%, Merck, (Lisboa, Portugal), CH_3_CO_3_H); ethanol (purity ≥ 99%, Valente e Ribeiro, (Alcanena, Portugal), C_2_H_5_OH); ammonium hydroxide (25% NH_3_ in H_2_O, Fisher Chemical, (Porto Salvo, Portugal), NH_4_OH); poly-ε-caprolactone (PCL, Mn 80,000, Sigma-Aldrich Chemicals, (Sintra, Portugal)); and tetrahydrofuran (THF, Sigma-Aldrich, (Sintra, Portugal)) were used. Dulbecco’s Modified Eagle’s Medium F12 (DMEM-F12) and resazurin were acquired from Sigma-Aldrich (Sintra, Portugal). Cell culture plates and T-flasks were purchased from Thermo Fisher Scientific (Porto, Portugal). Fetal Bovine Serum (FBS) was purchased from Biochrom AG (Berlin, Germany). Normal human osteoblast (hOB; 406-05f) cryopreserved cells were bought from Cell Applications, Inc. (San Diego, CA, USA).

### 3.2. Synthesis of the Aerogels

**Scheme 1:**
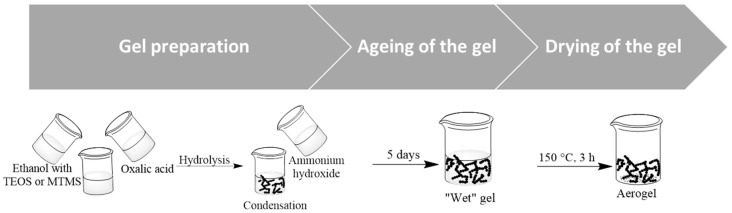
Schematic representation of sol–gel synthesis procedure.

The silica aerogel synthesis was prepared by the sol–gel method following a two-step acid-base catalyzed process previously reported [57,58] and schematically shown in Scheme 1. Shortly, in the first step, the precursors (TEOS or MTMS) were mixed with ethanol (solvent to Si molar ratio: S = 15 for TEOS and S = 25 for MTMS). Oxalic acid was added to catalyze the hydrolysis and the mixture was stirred for 30 min. The silica gel was obtained by a low-temperature sol–gel process. The nanostructured solid network of silica is formed as a result of hydrolysis and condensation reactions of the silica precursors, in which siloxane bridges (Si–O–Si) are formed. The hydrolysis step involved the conversion of the alkoxide to silanol. After 24 h of hydrolysis, a basic solution, NH_4_OH 2.5 M, was added to the former solution and kept under strong agitation for 1 min. The samples were kept in the oven at 27 °C for 5 days, for aging. 

Finally, the gels were dried at ambient pressure using an oven at 150 °C, for 3 h. After drying, the aerogel was ground, then sieved in order to obtain 75 μm sized grains.

### 3.3. Synthesis of the TEOS/PCL and MTMS/PCL Composites

**Scheme 2:**
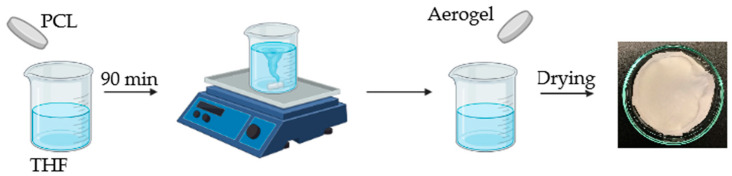
Schematic representation of composites synthesis procedure.

Initially, 1 g of PCL was dissolved in 10 mL of tetrahydrofuran (THF), under magnetic stirring for 90 min, followed by mixing with silica aerogel (<0.75 mm). The mixture was then casted into Petri dishes at room temperature. After evaporation of the solvent overnight at room temperature, the silica aerogel/PCL membranes were placed under vacuum for 5 h at room temperature to remove any traces of THF. Scheme 2 shows a representation of the procedure.

TEOS/PCL and MTM/PCL composites were fabricated at silica aerogel (TEOS or MTMS) to PCL (Mn 80,000) wt/wt ratios of 0.25:1 or 0.5:1.

The different composites where prepared with a diameter of 55 mm. Their different composition and aspect are given in Table 5.

### 3.4. Characterization of the Composites

The properties of aerogel, PCL, and final composites were assessed by different characterization techniques. 

The chemical structure was evaluated by attenuated total reflection (ATR) Fourier-transform infrared spectroscopy (FTIR) (FT/IR 4200, Jasco, Tokyo, Japan), collecting the spectra between a wavenumber of 4000 and 500 cm^−1^, with 128 scans and 4 cm^−1^ of resolution. 

Specific surface area (S_BET_) was assessed through nitrogen adsorption–desorption and the Brunauer–Emmet–Teller (BET) model (ASAP 2000, Micrometrics, Norcross, GA, USA).

Scanning electron microscopy (SEM) images were obtained using a Compact/VPCompact FESEM (Zeiss Merlin, Leipzig, Germany) microscope, after coating the samples with a thin gold layer by Physical Vapor Deposition, during 20 s. Two types of surfaces (surface and cross section from nitrogen rupture) were obtained scanned by SEM. The chemically of microstructure was characterized using energy-dispersive X-ray spectroscopy (EDS) (XMaxN, Oxford, UK).

The materials’ surface hydrophobicity was determined through contact angle measurements, using an OCA 20 system (Dataphysics, Filderstadt, Germany), with milliQ ultrapure water, at room temperature, by the sessile drop method. 

The bulk density (*ρ*_b_) was determined from the weight and volume of regular pieces of the samples. 

Thermal properties were assessed by thermal gravimetric analysis (TGA). The thermal stability of different prepared materials was obtained by using a DSC/TGA equipment (TGA-Q500, TA Instruments, New Castle, DE, USA), from 20 °C to 800 °C, at a 10 °C·min^−1^ heating rate under nitrogen flow. 

Static mechanical tests were also performed using an Inspekt mini-series equipment, from (Hegewald and Peschke, Nossen, Germany) at room temperature (20 °C). Uniaxial tension of the samples was performed with a load cell of 30 kN. The mechanical properties of the rectangular-shaped specimens (50 × 25 mm) were measured, and the crosshead speed was 0.1 mm/min. The tensile strength (*σ*, Mpa) and the elongation at break (*ε*) were calculated according to Equations (1) and (2), respectively:

(1) $$\sigma =\frac{F}{A}$$

(2) $$\varepsilon =\frac{∆l}{l_{o}}\times 100$$

where *F* is the maximum force at break, *A* is the cross-sectional area of the sample, *l*_0_ is the initial distance between the texturometer grips (5 cm), and Δ*l* is the distance different between *l_0_* and the distance of the grips at the time of sample break. The modulus of elasticity (Young’s modulus, E’) was defined as the slope of linear section of the Max *σ* versus *ε* curve, being expressed in MPa.

In order to determine the swelling capacity, three quarters of each prepared adhesive was dried under vacuum conditions until constant weight (W_d_) and then placed in a desiccator with a saturated solution of pentahydrated copper sulphate. All the samples were weighted at predetermined times until a maximum weight was achieved (W_s_) in a Sartorius Electronic Balance (BCE223I-1SJP, Entris II, read limit: 1 mg, maximum capacity: 220 g, Goettingen, Germany) and water sorption (%) was calculated by using Equation (3).

(3) $$\mathrm{Swelling}\;\left(\%\right)=\left(\frac{W_{s}-W_{d}}{W_{d}}\right)\;\times 100$$

Degradation was evaluated for three samples of each material, which were dried until constant weight and then weighted (W_d,0_); afterwards, they were immersed of phosphate buffer solution 0.01 M (pH = 7.4), and then incubated at 37 °C for five weeks. The samples were removed from PBS, washed with distilled water, and dried under vacuum, until constant weight (W_d,t_) in a Sartorius Electronic Balance (BCE223I-1SJP, Entris II, read limit: 1 mg, maximum capacity: 220 g, Goettingen, Germany). The relative mass loss was evaluated according to Equation (4):

(4) $$\mathrm{Mass}\;\mathrm{loss}\;\left(\%\right)=\frac{W_{d,0}-W_{d,t}}{W_{d,0}}\;\times 100$$

where W_d,0_ and W_d,t_ are the samples mass before the immersion in PBS and after the immersion at time t, respectively.

The cytocompatibility of the produced materials towards hOB cells was evaluated through the resazurin assay [59,60]. The hOB cells were cultured in DMEM-F12 supplemented with 10% (*v*/*v*) of FBS and 1% (*v*/*v*) of penicillin/streptomycin in an incubator with humidified atmosphere (37 °C, 5% CO_2_). For the cytocompatibility assay, the hOB cells were seeded at specific densities in 96-well plates, according to the total incubation time with the materials, to avoid cells’ death by confluence. In this regard, 20 × 10^3^, 10 × 10^3^, and 8 × 10^3^ cells/well were initially seeded for the groups intended to be incubated with the silica aerogel-PCL scaffolds for 1, 3, and 7 days, respectively. 

After 24 h of hOB cells’ seeding, these were incubated with M0.25_PCL1, M0.5_PCL1, T0.25_PCL1, and T0.5_PCL1 (previously cut into small pieces with sizes bellow 10% of the well area) along with fresh culture medium for 1, 3, and 7 days. After the incubation periods, the silica aerogel-PCL scaffolds were removed, and the hOB cells were put in contact with fresh culture medium containing resazurin (10% (*v*/*v*)) for 4 h in the dark (37 °C, 5% CO_2_). Then, the hOB cells’ viability was assessed by analyzing the fluorescence of resorufin (λ_ex_/λ_em_ = 560/590 nm; Spectramax Gemini EM spectrofluorometer). hOB cells solely incubated with culture medium were used as the negative control (K−) while those exposed to ethanol (70% (*v*/*v*)) were the positive control (K+). Throughout this assay, the hOB cells’ growth/morphology was also monitored by using an Olympus CX41 inverted light microscope (Tokyo, Japan) equipped with an Olympus SP-500 UZ digital camera. 

## 4. Conclusions

In this work, scaffolds assembled with PCL and two silica-based aerogels, one hydrophilic (TEOS-aerogel based) and other hydrophobic (MTMS-aerogel based), have been successfully prepared, characterized, and compared. MTMS-aerogel composite presented higher density, specific surface area, and hydrophobicity than TEOS-aerogel composites, due to the presence of -CH_3_ groups, and by the more porous surface shown in SEM images. SEM images and EDS analysis also indicated that the silica was well incorporated with the polymer. The Young’s modulus of the obtained hybrid scaffolds increased with decreasing the amount of aerogel and MTMS-aerogel composites were less rigid than TEOS-aerogel composites. The scaffolds also showed low swelling (maximum of 14%) and low mass loss. However, TEOS-based scaffolds, which were slightly less hydrophobic, showed higher mass loss and higher swelling than MTMS-based scaffold. In the in vitro studies, the scaffolds presented a good cytocompatibility up to 7 days.

Overall, the developed hybrid scaffolds may be promising structures for tissue engineering applications. In the future, in vitro tests, such as the evaluation of antimicrobial and in vivo tests, can be considered to further characterize these hybrid scaffolds.

## Figures and Tables

**Table 1:** BET specific surface area (m^2^g^−1^) of the hybrid scaffolds and PCL.

Composites	BET Specific Surface Area (m^2^g^−1^)
M0.5_PCL1	27.84 ± 0.64
M0.25_PCL1	28.78 ± 0.88
T0.5_PCL1	4.04 ± 0.18
T0.25_PCL1	2.88 ± 0.20
PCL	16.28 ± 0.52

**Table 2:** Static water contact angle of different composites (*n* = 4).

Composites	Water Contact Angle (°)
M0.5_PCL1	98.5 ± 1.0
M0.25_PCL1	98.2 ± 1.5
T0.5_PCL1	90.3 ± 1.1
T0.25_PCL1	91.8 ± 0.9

**Table 3:** Bulk density for different composites (*n* = 4).

Samples	Density (kg/m^3^)
M0.25_PCL1	505.968 ± 0.647
M0.5_PCL1	361.257 ± 0.893
T0.25_PCL1	365.903 ± 0.928
T0.5_PCL1	171.371 ± 1.754

**Table 4:** Measured mechanical properties of the assessed samples (*n* = 4).

Composites	Young’s Modulus (MPa)	Maximum Stress (MPa)
M0.5_PCL1	15.84 ± 1.03	39.68 ± 4.73
M0.25_PCL1	19.20 ± 3.35	48.70 ± 3.41
T0.5_PCL1	28.01 ± 1.84	71.60 ± 0.40
T0.25_PCL1	37.07 ± 2.32	108.86 ± 0.51
PCL	12.77 ± 0.63	58.10 ± 1.99

**Table 5:** Samples’ nomenclature and composition.

Samples	Images	Composition
M0.5_PCL1	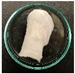	0.5 g Aerogel MTMS + 1 g PCL
M0.25_PCL1	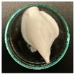	0.25 g Aerogel MTMS + 1 g PCL
T0.5_PCL1	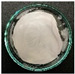	0.5 g Aerogel TEOS + 1 g PCL
T0.25_PCL1	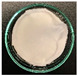	0.25 g Aerogel TEOS + 1 g PCL
PCL	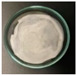	1 g PCL

## Data Availability

Data is contained within the article. The raw/processed data required to reproduce these findings cannot be shared at this time due to technical or time limitations, but will be sent upon request.
